# Supplementary material for: Cohesin occupancy and composition at enhancers and promoters are linked to DNA replication origin proximity in Drosophila
Source: Genome Res. 2019 Apr;29(4):602–12. doi: 10.1101/gr.243832.118 (PMC6442380; doi:10.1101/gr.243832.118)
Supplement: Supplemental Material [file supp_29_4_602__index.html]

Cohesin occupancy and composition at enhancers and promoters are linked to DNA replication origin proximity in Drosophila — Supplemental Material 

# Cohesin occupancy and composition at enhancers and promoters are linked to DNA replication origin proximity in *Drosophila*

## Supplemental Material

- Supplemental\_Material.rev.pdf
